# Chagasic patients are able to respond against a viral antigen from influenza virus

**DOI:** 10.1186/1471-2334-12-198

**Published:** 2012-08-24

**Authors:** Paola Lasso, Diana Mesa, Natalia Bolaños, Adriana Cuéllar, Fanny Guzmán, Zulma Cucunuba, Fernando Rosas, Víctor Velasco, Maria C Thomas, Manuel Carlos López, John Mario González, Concepción Judith Puerta

**Affiliations:** 1 Laboratorio de Parasitología Molecular, Pontificia Universidad Javeriana, Carrera 7 No. 43 – 82, Bogotá, Colombia; 2 Grupo de Ciencias Básicas Médicas, Facultad de Medicina, Universidad de los Andes, Carrera 1 No. 18A – 10, Bogotá, Colombia; 3 Grupo de Inmunobiología y Biología Celular, Pontificia Universidad Javeriana, Carrera 7 No. 43 – 82, Bogotá, Colombia; 4 Núcleo de Biotecnología Curauma, Pontificia Universidad Católica de Valparaíso, Avenida Universidad 330, Valparaíso, Chile; 5 Grupo de Parasitología, Instituto Nacional de Salud, Avenida Calle 26 No. 51 – 20, Bogotá, Colombia; 6 Fundación Clínica Abood Shaio, Diag. 115A No. 70C – 75, Bogotá, Colombia; 7 Instituto de Parasitología y Biomedicina López Neyra, Consejo Superior de Investigaciones Científicas (IPBLN-CSIC), Parque Tecnológico de Ciencias de la Salud, Avda. del Conocimiento, s/n.18100, Granada, Spain; 8 Departamento de Microbiología, Facultad de Ciencias, Laboratorio de Parasitología Molecular, Pontificia Universidad Javeriana, Bogotá, Colombia

**Keywords:** CD8^+^ T cells, Chagas disease, Non-*T. cruzi* microbial antigen, Nonspecific immune-suppression

## Abstract

**Background:**

*Trypanosoma cruzi,* the etiological agent of Chagas’ disease*,* is an obligate intracellular parasite which induces a CD8^+^ T cell immune response with secretion of cytokines and release of cytotoxic granules. Although an immune-suppressive effect of *T. cruzi* on the acute phase of the disease has been described, little is known about the capacity of CD8^+^ T cell from chronic chagasic patients to respond to a non-*T. cruzi* microbial antigen.

**Methods:**

In the present paper, the frequency, phenotype and the functional activity of the CD8^+^ T cells specific from Flu-MP*, an influenza virus epitope, were determined in 13 chagasic patients and 5 healthy donors.

**Results:**

The results show that Flu-MP* peptide specific CD8^+^ T cells were found with similar frequencies in both groups. In addition, Flu-MP* specific CD8^+^ T cells were distributed in the early or intermediate/late differentiation stages without showing enrichment of a specific sub-population. The mentioned Flu-MP* specific CD8^+^ T cells from chagasic patients were predominately T_EM_ (CCR7- CD62L-), producing IL-2, IFNγ, CD107a/b and perforin, and did not present significant differences when compared with those from healthy donors.

**Conclusions:**

Our results support the hypothesis that there is no CD8^+^ T cell nonspecific immune-suppression during chronic Chagas disease infection. Nonetheless, other viral antigens must be studied in order to confirm our findings.

## Background

Chagas’ disease, caused by the hemoflagellate parasite *Trypanosoma cruzi*, has a widespread distribution in Central and South America. Approximately 10 million people are infected along 21 endemic countries of Latin-America [1]. However, due to increasing migration to North America and Europe, the disease has been expanded beyond its original borders [2,3]. Infection with *T. cruzi*, results in a generally asymptomatic disease due to the fact that most of the infected individuals remains in an indeterminate chronic phase. After the acute phase, the disease becomes clinically evident in 30-40% of them with cardiac or digestive manifestations (symptomatic chronic phase) [4-7].

*Trypanosoma cruzi* is an obligate intracellular parasite which invades and replicates into mammalian cells. As other intracellular infectious agents, *T. cruzi* induces a CD8^+^ T cell immune response with secretion of cytokines and release of cytotoxic granules [8,9]. The role of CD8^+^ T cells is crucial for controlling the *T. cruzi* infection. Indeed, during the acute infection, depleted CD8^+^ T cells mice showed increased parasite burden in their hearts, moderate decrease in the inflammation and higher mortality compared with wild type infected animals [10]. Nonetheless, in chronic infection, there are several experimental data supporting that the suboptimal generation of functional specific effector CD8^+^ T cells leads to the lack of infection control and parasite persistence in the tissues [11,12]. Thus, the presence of late-differentiated CD8^+^ T cells exhibiting signs of senescence that are incapable of maintaining effector functions [11-13], and the diversity of *T. cruzi* antigens that can enter MHC class I processing pathway should be responsible of the absence of a potent and antigen focused cellular immune response [8,14]. Due to the parasite’s genetic complexity and a high degree of polymorphism among strains, only a few human CD8^+^ T cell parasite-specific epitopes capable of inducing specific CD8^+^ T cell responses have been described [14-21]. Furthermore, even less is known about the capacity of chagasic patients to respond to non-chagasic microbial antigens.

A *T. cruzi*-induced nonspecific immune-suppression during the acute phase of the disease has been reported by several authors, showing the immunosuppressive effect on dendritic cells, macrophages, splenic T cells, and LTs [22-25]. Nonetheless, experimental infection in mice indicated that there is no general immunosuppressive effect of *T. cruzi* on CD8^+^ T cell priming [26]. No data of CD8^+^ T cell immune-suppression have been reported with another derived-microbial epitopes in humans infected with *T. cruzi*.

The aim of the present work was to determine whether or not there is a nonspecific immune-suppression in chronic chagasic patients. Consequently, we investigated the CD8^+^ T cells response against a representative viral antigen. Thus, we examined the frequency, phenotype and the functional activity of the influenza virus specific CD8^+^ T cells in chagasic patients and healthy donors. We selected an epitope from the viral matrix protein because the majority of individuals throughout their life have been exposed to the influenza virus and have had at least one infection.

## Results

### Population HLA-A2 subtyping

In this work we assessed whether the CD8^+^ T cells from *T. cruzi* infected patients are able to respond to non-*T. cruzi* microbial antigens such as a well described epitope from influenza virus derived from the matrix protein. Specifically, it was studied whether the frequency, phenotype and functional capacity of Flu-specific CD8^+^ T cells are modified in chronic chagasic patients with respect to healthy donors used as control. The Flu-MP* peptide, which is an HLA-A*0201 restricted epitope was used as non-*T cruzi* epitope since HLA-A*0201 is the most prevalent human class I allele [27]. Consequently, subjects were HLA-A2 typed by flow cytometry and subtyped for HLA-A*0201 by PCR. It was found that 13 out of 50 chagasic patients (26%) were HLA-A2^+^; from whom all were HLA-A*0201^+^, classified as six chronic patients in indeterminate phase or A stage (normal electrocardiography ECG, and no major findings arising during clinical examination) and seven symptomatic chagasic patients having different disease severity levels as follows: 2 B (abnormal ECG results), 3 C (abnormal ECG results, decreased left ventricular ejection fraction, LVEF, and cardiac enlargement) and 2 D (abnormal ECG results, cardiac enlargement, decreased LEFV, and clinical signs of heart failure). Analysis of the twelve healthy donors showed that five of them (41.7%) were HLA-A2^+^, and HLA-A*0201^+^.

### Frequency of MP-Flu* specific CD8^+^ T cells

**Figure 1:**
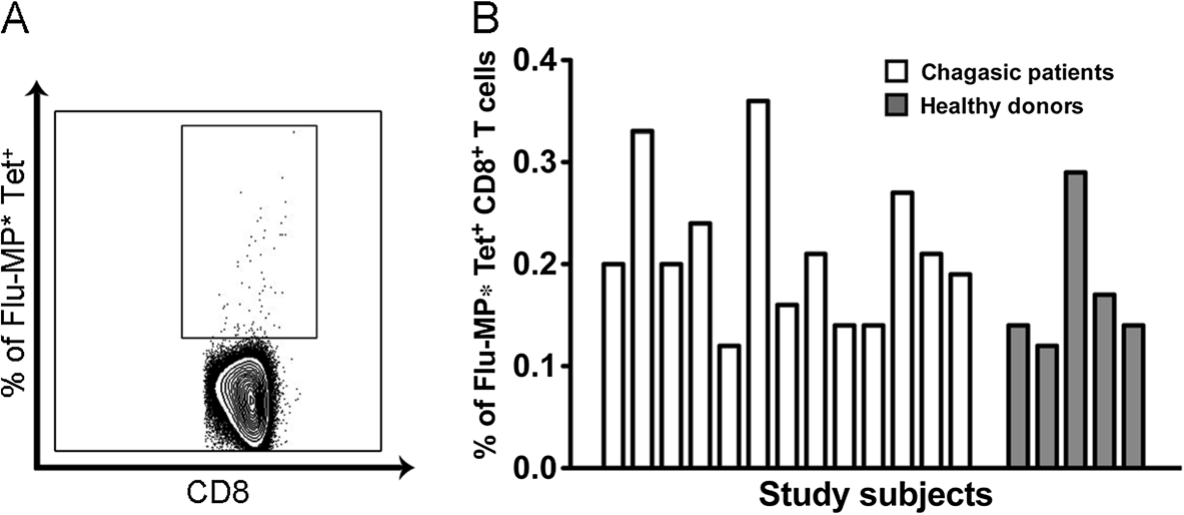
**Frequency of CD8**^**+**^T cells specific for Flu-MP* peptide determined by tetramer staining. (**A**) Representative flow cytometry dot plot from one HLA-A*0201^+^ chagasic patient. (**B**) Frequency of HLA-A*0201^+^ chagasic patients (white bars) and HLA-A*0201^+^ healthy donors (grey bars). There were no statistically significant differences between healthy donors and chagasic patients *P* = 0.21.

Tetramer consisting of HLA-A*0201/Flu-MP* peptide was used to evaluate the frequency of Flu-MP* specific CD8^+^ T cells in the thirteen HLA-A*0201^+^ chagasic patients and five HLA-A*0201^+^ healthy donors. Flu-MP* peptide specific cells were found in all chagasic patients, showing a range of frequency of 0.12% to 0.36% (mean 0.21%, SD = 0.07). Likewise, in the group of healthy donors all individuals had Flu-MP* peptide specific cells with a frequency range of 0.12% to 0.29% (mean 0.17%, SD = 0.07). There was no difference when all the groups of patients were compared based on their clinical status (indeterminate and cardiac chronic patients) or when chagasic patients were compared with healthy donors (P >0.05) (Figure 1).

### Phenotypic characterization of Flu-MP* specific-CD8^+^ T cells

**Figure 2:**
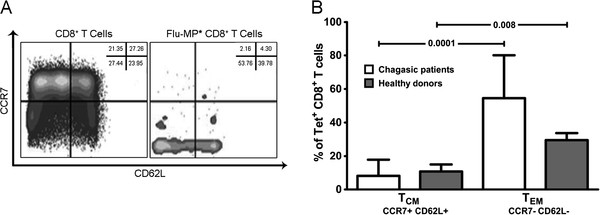
Expression of CCR7 and CD62L in Flu-MP* peptide-specific CD8^**+**^**T cells.** (**A**) Phenotypic characterization by flow cytometry in one representative chagasic patient. (**B**) Significant differences were observed between central memory (double positive cells) T cells (T_CM_) and effector memory (double negative cells) T cells (T_EM_) (*P* < 0.008) from chagasic patients (white bars) or healthy donors (grey bars). No differences were found when the frequencies of T_CM_ and T_EM_ CD8^+^ T cells were compared between chagasic patients and healthy donors.

**Figure 3:**
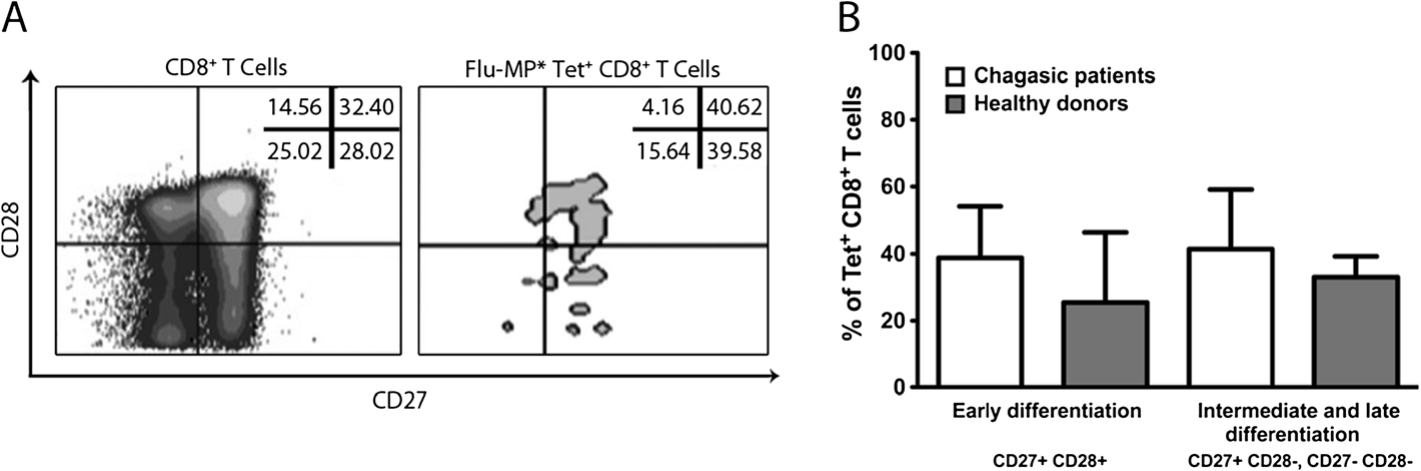
Expression of CD27 and CD28 in Flu-MP* peptide-specific CD8^**+**^**T cells.** (**A**) Flow cytometry dot plot of CD27 and CD28 expression in one representative chagasic patient. (**B**) There were no differences between the early differentiation phenotype (CD27+ CD28+) and the intermediate/late differentiation stage (CD27+ CD28-, CD27- CD28-) in CD8^+^ T cells (*P* > 0.151). There were no significant differences between the chagasic patients (white bars) and healthy donors (grey bars) when comparing the early phenotype (*P* = 0.139) with intermediate/late differentiation stage (*P* = 0.430).

Several studies showed the importance of memory CD8^+^ T cells in controlling viral infections in mice and human models [28]. Consequently, the phenotype of the Flu-MP* specific CD8^+^ T cells from HLA-A*0201^+^ infected patients and healthy donors was determined by analyzing the expression of CCR7, CD62L, CD27 and CD28. It was found that the Flu-MP* peptide-specific CD8^+^ T cells were predominately T_EM_ (CCR7- CD62L-) in both indeterminate and cardiac chronic chagasic patients without statistically differences (P = 0.206). Moreover, no differences were found when the expression in chagasic patients and healthy donors were compared (P = 0.055). However, significant differences were observed when the percentages of Flu-MP* peptide specific T_CM_ and T_EM_ cells from chagasic patients or healthy donors (P = 0.0001, and P = 0.008, respectively) were compared (Figure 2). As the CD8^+^ T cell differentiation degree, based on CD28, and CD27 expression, well correlates with their functional capacity [29], the Flu-specific CD8^+^ T cell stage of differentiation was compared among donors. It was found that Flu-MP* peptide-specific CD8^+^ T cells are equally distributed in the early and intermediate/late differentiation stages without showing any enrichment of a specific population. There were no statistically significant differences between chagasic patients and healthy donors (Figure 3). 

### Functional characterization of Flu-MP* specific-CD8^+^ T cells

**Figure 4:**
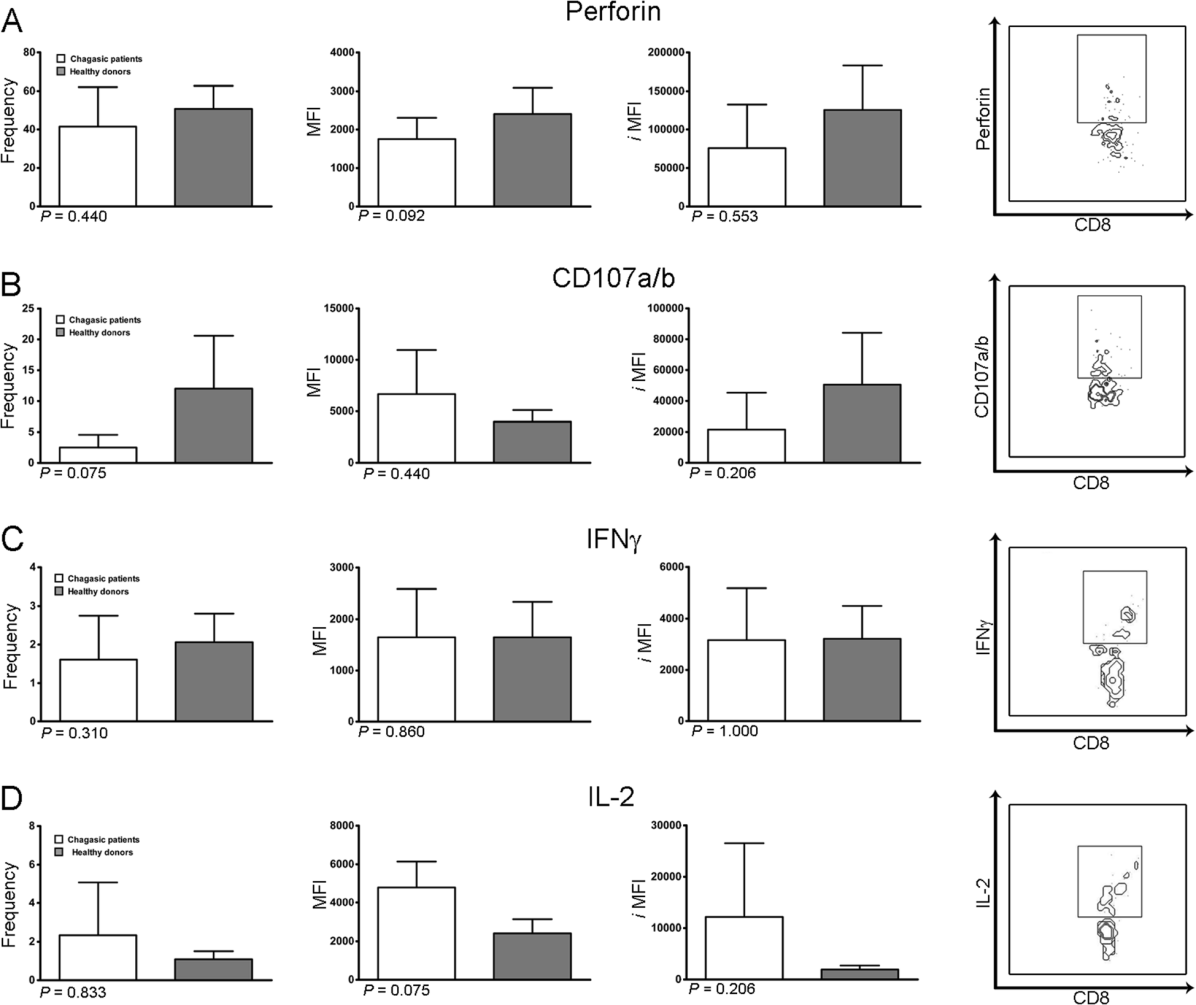
**Functional characterization of CD8**^**+**^ T cells specific for Flu-MP* peptide determinated by flow cytometry.  Frequency, mean fluorescence intensity (MFI) and integrated mean fluorescence intensity (*i*MFI) of the perforin (**A**), CD107a/b (**B**), IFNγ (**C**) and IL-2 (**D**) expression in CD8^+^ T cells specific for Flu-MP* peptide in chagasic patients (white bars) and healthy donors (grey bars). A representative dot plot of the expression of each studied molecule from one HLA-A*0201^+^ chagasic patient is shown on the right side.

To assess the functional characteristics of Flu-MP* specific CD8^+^ T cells, the IL-2 and IFNγ cytokine production and cytotoxic capacity, measured by the perforin secretion and CD107a/b surface expression, were evaluated in PBMC from ten chagasic patients and 5 healthy donors. For perforin analysis, it was observed that Flu-MP* peptide specific CD8^+^ T cells from *T. cruzi*-infected volunteers had similar percentage of expression, ranging from 17.56% to 77.78% (mean 41.4%, SD = 20.60%), than that from the healthy group of donors which ranged from 30.3% to 61.5% (mean 50.62%, SD = 12.05%). The surface expression of CD107a/b was detected in Flu-MP* peptide specific CD8^+^ T cells from both chagasic patients and healthy donors, with percentages ranging from 0.52 to 5.71% (mean 2.50%, SD = 2.05%) and 0.47% to 24.2% (mean 12.01%, SD = 8.57%), respectively. Furthermore, no differences in percentage, mean fluorescence intensity (MFI) or integrated mean fluorescence intensity (*i*MFI) in CD107a/b between chagasic patients and healthy donors were observed (Figure 4A, 4B). IL-2 was detected in eight out of ten infected patients with a range from 0.21% to 7.37% (mean 1.87%, SD = 2.6%) and in all studied healthy donors with a range from 0.56% to 1.57% (mean 1.09%, SD = 0.42%). IFNγ production was detected in Flu-MP* specific CD8^+^ T cells with a range from 0.51% to 3.42% (mean 1.61%, SD = 1.13%) in nine out of ten chagasic patients and in all healthy donors with a frequency range of 1.33% to 3.07% (mean 2.06%, SD = 0.74%). Consequently, no differences in cytokines production were observed in terms of percentage, MFI or *i*MFI when both groups were compared (Figure 4C, 4D).

## Discussion

Chagas disease is a persistent parasitic infection that does not induce sterilizing immunity. The generation of memory T cell response depends on the environmental conditions at the initial antigenic priming [30]. Indeed, the T cell responses may be influenced by previous antigen encounters, a phenomenon known as “original antigenc sin” [31]. Thus, the goal of this study was to determine whether chagasic patients are able to respond against a non-*T. cruzi* microbial antigen such as the matrix peptide derived from influenza virus. Exposure to influenza virus is widely spread therefore most individuals develop a serotype-dependent sterilizing immunity.

At first, frequency of Flu-MP* peptide-specific CD8^+^ T cells from chronic chagasic patients and healthy donors was studied. Noticeably, no differences were found between these two groups. Moreover, the average frequency values were similar to those previously reported for the influenza-M1_58–66_ (GILGFVFTL) specific CD8^+^ T cells (0.28% to 0.73%) from healthy donors [32]. A similar Flu-MP tetramer positive cell frequency (0.11% to 0.56%) was reported in six HLA-A*0201+ healthy donors [33]. Likewise, in a previous study we reported the existence of Flu-MP*-specific CD8^+^ T cells with frequencies up to 0.36% in 16 out of 19 HLA-A2+ chagasic patients and frequencies up to 0.25% in 10 out of 12 healthy donors without statistically significant difference when non-chagasic and *T. cruzi* infected individuals were compared (P > 0**.**05) [34].

Memory T cells consist in central (T_CM_) and effector (T_EM_) memory cells [28,30,35]. While human T_CM_ express CCR7 and CD62L, molecules involved in cellular homing to lymphoid tissue, human T_EM_ have lost the expression of CCR7 and CD62L and preferentially migrate to non-immune tissues [28]. In this study, Flu-MP* peptide-specific CD8^+^ T cells were predominately T_EM_ cells, in both healthy donors and chagasic patients. Results that are in agreement with reports of Hoji et al., showing that Flu-specific T cells from healthy donors had a lower proportion of CCR7 expression on Flu-specific T cells and a moderate expression of CD62L with a phenotype that corresponds to effector memory CD8^+^ T cells [33]. Expression of CD27 and CD28 are useful in distinguishing CD8^+^ T cell differentiation stages: early (CD27+ CD28+), intermediate (CD27+ CD28-) and late differentiation (CD27- CD28-) [28]. In our study, in the influenza-specific CD8^+^ T cells there was not a predominant population based on CD27 and CD28 expression. During chronic viral infection, and depending on the infecting virus, there is a certain predominance of a cell stage of differentiation [29]. Nonetheless, we could not find any report in the literature showing the expression of these markers in influenza-specific CD8^+^ T cells to be compared with our results.

CD8^+^ T_EM_ cells have cytotoxic granules and produce cytokines within hours after antigenic stimulation. MP-Flu* peptide-specific CD8^+^ T cells expressed surface CD107a/b and were able to secrete IL-2, IFNγ and storage perforin. Interestingly, in both infected patients and healthy donors, the expression of IL-2, IFNγ and perforin by Flu-MP* peptide-specific CD8^+^ T cells was similar in magnitude (percentage of expressing cells), quality (MFI) or both (iMFI), indicating that these populations are equally functional upon Flu-MP* peptide recognition.

In agreement with results presented here, a study of congenital Chagas disease performed in newborns congenitally infected with *T. cruzi*, showed that the infection did not interfere with responses to Bacillus Calmette Guerin (BCG), hepatitis B, diphtheria, and tetanus vaccines during the neonatal period [36].

## Conclusion

Our results support the hypothesis that there is no CD8^+^ T cell nonspecific immune-suppression during chronic Chagas disease infection. Nonetheless, other viral antigens must be studied in order to confirm our findings.

## Methods

### Subjects

All individuals enrolled in the study were volunteers who signed the informed consent form and were clinically evaluated at Fundación Abood Clínica Shaio and the Instituto Nacional de Salud (Bogotá, Colombia). Fifty Chagas disease patients with positive results in both immunofluorescence assays (IFI) and enzyme-linked immunosorbent assays (ELISA) were evaluated. Patients were classified according to the classification of the Incorporating American College of Cardiology and the American Heart Association Staging [37,38], as follows: twenty-one chronic chagasic patients at indeterminate phase of the disease with normal ECG and no major findings arising during clinical examination (group A), and twenty nine cardiac chronic chagasic patients with abnormal ECG, having different disease severity levels (groups B, C and D). The group of healthy donors consisted of twelve *T. cruzi* seronegative individuals residing in non-endemic areas with normal ECG and no major clinical findings during clinical examination. Approximately a total of 20 mL of blood was obtained from all individuals by venipuncture in heparinized tubes to separate peripheral blood mononuclear cells (PBMCs), EDTA tubes for DNA extraction and tubes without anticoagulant for serological tests (Vacutainer; Beckton-Dickinson, San José, CA, USA). This study was approved by the Research and Ethics Committees from the Facultad de Ciencias, Pontificia Universidad Javeriana and Fundación Abood Clínica Shaio.

### Flu-MP* modified peptide

An HLA-A*0201 restricted peptide derived from the influenza virus matrix protein_58-66_ with a modification in the seventh position was used (GILGFVTTL) and referred as Flu-MP* [19,34,39]. Peptide was synthesized using 9-fluorenylmethiloxycarbonyl (Fmoc) chemistry with a 0.64 substituted rink amide resin and Fmoc amino acids [40]. The peptides were cleaved by treatment with trifluoroacetic acid (TFA)/triisopropylsilan (TIS)/H2O (95/5/5) for 1 h, then precipitated with cold diethyl ether and purified by reverse phase high performance liquid chromatography (HPLC). The molar mass of the peptides was determined by MALDI-TOF mass spectrometry. The lyophilized peptide was reconstituted in dimethyl sulfoxide (DMSO) and stored at −20°C.

### HLA-A2 typing by flow cytometry

Individual HLA-A2 typing was performed by incubating 50 μL of peripheral blood with HLA-A2-specific antibody BB7⋅2-FITC (Becton Dickinson, Mountain View, California, USA) for 20 min at 4°C, followed by additional 15 min incubation. After adding red blood cells lysis buffer and 1X PBS washing [41], data were acquired and analyzed using a FACSCanto II flow cytometer and analyzed using FACSDiva (BD Biosciences) software.

### HLA-A*0201 subtyping by PCR

A total of 50–150 ng genomic DNA was extracted from peripheral blood using a GFX genomic blood DNA purification kit (Amersham Biosciences, Piscataway, NJ, USA), and amplified using the 296 (5′-GTGGATAGAGCAGGAGGCT-3′), and 302 (5′-CCAAGAGCGCAGGTCCTCT-3′) primers. The PCR reaction was carried out according to Lasso et al. 2010 in a Stomacher 3500 Thermal Cycler, PTC 100 (MJ Research, Watertown, MA, USA) [34]. The expected 489 bp amplified product was separated on ethidium bromide stained 1.5% agarose gels [42].

### T cell frequency determination

HLA-A2 PE-labeled tetramer loaded with the Flu-MP* peptide, was synthesized by the National Institute of Health (NIH) Tetramer Facility (Atlanta, USA). Peripheral blood mononuclear cells (PBMCs) of all individuals were prepared using Ficoll-Hypaque density gradient (Sigma, St. Louis, MO), adjusted to 1 x 10^6^ cells per tube and stained with tetramer at 0,5 μg/mL, anti-CD3-PerCP and anti-CD8-FITC (BD Biosciences, San José, CA, USA) for 20 min in the dark at room temperature [34]. After washing with staining buffer (1% fetal bovine serum in PBS 1X), cells were resuspended with 500 μL of PBS 1X. Data were acquired in a FACSCanto II flow cytometer and analyzed using FACSDiva software (BD, Bioscience, San José, CA, USA). Frequencies of Flu-specific CD8^+^ T cells are the result of the frequency obtained minus the background of unlabeled cells with the tetramer.

### T cell phenotyping and function

PBMCs were adjusted to 2 x 10^6^ cells per tube and labeled with tetramers at 0.5 μg/mL, anti-CD3-PerCP, anti-CD8-APC-Cy7, anti-CCR7-PECy7, anti-CD62L-APC for 20 min in the dark at room temperature (all antibodies were purchased from BD Biosciences and BD Pharmingen, San Diego, CA, USA). After washing with 2 mL staining buffer (1% fetal bovine serum in PBS 1X), cells were fixed with 0.5% formaldehyde in PBS 1X. To determine intracellular perforin expression, after surface antigens staining, CD8^+^ T cells were permeabilized with Cytofix/Cytoperm (BD Pharmingen), washed twice with Perm/Wash^TM^ (BD Pharmingen) and stained with anti-perforin-FITC or mouse IgG2b κ isotype control for 30 min at 4°C. Intracellular cytokines were detected in PBMCs stimulated with Flu-MP* peptide in the presence of CD28 (1 μg/mL), and CD49d (1 μg/mL) for 12 h at 37°C. The last 9 h of culture were performed in the presence of brefeldin A (10 μg/mL) (BD Pharmingen). Then, cells were permeabilized and stained with anti-IFNγ-PECy7 and anti-IL-2-APC for 30 min at 4°C. To evaluate the cytotoxic activity, anti-CD107a and anti-CD107b-FITC were added to the PBMCs prior to stimulation. In each experiment, non-stimulated cells were used as negative control and Staphylococcal enterotoxin B (3.7 μg/mL) as positive control. Data were acquired in a FACSCanto II flow cytometer and analyzed using FACSDiva (BD Biosciences) software. Results were expressed as percentage, mean fluorescence intensity (MFI) and integrated mean fluorescence intensity (iMFI) obtained multiplying the frequency by the MFI. The latter was obtained multiplying the frequency by the MFI of tetramer positive CD8^+^ T cells [43].

### Statistical methods

Differences among groups were assessed by Mann Whitney test. GraphPad InStat 3.0 statistics software was used for analyses. P < 0.05 was considered statistically significant.

## Competing interests

The authors declare that they have no competing interests.

## Authors’ contribution

PL performed the experiments, participated in the data analysis and wrote the manuscript. DM and NB performed the experiments and participated in the data analysis. AC and JMG participated in the designed the study, analyzed the data and collaborated writing the manuscript. ZC, VV and FR recruited and assessed the patients and collected the clinical information. MCT, MCL and FG participated in the data analysis and collaborated writing the manuscript. CPB conceived and designed the study, obtained financial support, analyzed the data and wrote the manuscript. All authors participated in revising the manuscript. All authors have read and approved the final manuscript.

## Pre-publication history

The pre-publication history for this paper can be accessed here:

http://www.biomedcentral.com/1471-2334/12/198/prepub
